# Ensuring radiology reporting quality across a national lung cancer screening program

**DOI:** 10.1093/bjr/tqag158

**Published:** 2026-06-29

**Authors:** Judith L Babar, Ian Selby, Samanjit S Hare, Aniket Tavare, Jonathan C L Rodrigues

**Affiliations:** The HLH Imaging Group Ltd, Ruislip, Middlesex HA4 0BW, United Kingdom; Department of Radiology, Cambridge University Hospitals NHS Foundation Trust, Cambridge CB2 0QQ, United Kingdom; The HLH Imaging Group Ltd, Ruislip, Middlesex HA4 0BW, United Kingdom; The HLH Imaging Group Ltd, Ruislip, Middlesex HA4 0BW, United Kingdom; Department of Radiology, Royal Free London NHS Trust, London NW3 2QG, United Kingdom; The HLH Imaging Group Ltd, Ruislip, Middlesex HA4 0BW, United Kingdom; Department of Radiology, Northumbria Healthcare NHS Foundation Trust, North Shields, Tyne and Wear NE29 8NH, United Kingdom; The HLH Imaging Group Ltd, Ruislip, Middlesex HA4 0BW, United Kingdom; Department of Radiology, Royal United Hospital Bath NHS Foundation Trust, Bath BA1 3NG, United Kingdom; Department of Health, University of Bath, Bath BA2 7AY, United Kingdom

**Keywords:** lung cancer screening, quality assurance, CT scan reporting

## Abstract

Low-dose CT (LDCT) for lung cancer screening (LCS) in high-risk individuals reduces both lung cancer-specific and all-cause mortality, supported by data from the National Lung Screening Trial, NELSON, the International Early Lung Cancer Action Program (I-ELCAP). In 2019, following a successful pilot, the UK National Health Service announced rollout of the Targeted Lung Health Check (TLHC) program, offering ever-smokers aged 55-74 an appointment with a health professional, which may lead to referral for LDCT based on individual risk of developing lung cancer assessed using validated multivariable risk-prediction models (PLCOm2012 and the Liverpool Lung Project model, LLPv2). As LCS expands, it is paramount that radiologists who participate in screening are adequately qualified and properly trained. To ensure the radiology reporting for an expanding program remains clinically and cost effective, reporters must engage with nationally-set quality assurance (QA) policies. In this article, we describe the approach taken by The HLH Imaging Group (HLH), a provider reporting ∼85% of LCS scans in NHS England (NHSE), to embed near real-time and routine double-read QA alongside the national external quality assurance (EQA) scheme (PERFECTS). We present aggregate referral-rate data from reporters in our network across the 2025-26 financial year and compare current NHSE fixed thresholds, with statistically-derived thresholds (mean ± 2SD; median ± *k*·MAD). We provide explicit rationale for our operational choices (a 2-consecutive-quarter outlier definition, 25-case retrospective review of flagged reporters, and 5% random sampling of negative reports for blinded double reading). We explicitly acknowledge that this describes a provider’s approach within a wider radiological workforce; PERFECTS remains the only program-wide QA covering all NHSE LCS readers. We propose that the elements described could form the basis of an NHSE/Department of Health and Social Care standardized near real-time QA framework applicable across all LCS providers.

## Evidence for lung cancer screening

Lung cancer is the leading cause of cancer death worldwide, with an age-standardized incidence rate (ASR) of approximately 23.6 per 100 000 and an age-standardized mortality rate of 16.8 per 100 000 in 2022 (GLOBOCAN).^1^^,^^2^ Due to the high socioeconomic burden of late-stage lung cancer diagnosis, early observational studies of lung cancer screening (LCS) using chest radiograph (CXR) and sputum cytology were initiated in the 1950s, with the first randomized controlled trials of CXR-based screening (including the Mayo Lung Project, the Memorial Sloan-Kettering, and Johns Hopkins studies) initiated in the 1970s.^3^ From the 1990s, new developments in computed tomography (CT) paved the way for low-dose CT (LDCT)-based LCS.^4^ Multiple randomized controlled trials have since demonstrated that LCS with LDCT reduces lung cancer-specific mortality through a stage shift in detection, with consequent higher rates of curative-intent treatment.^5^ The National Lung Screening Trial (NLST) in the United States used a 2-stage randomization process of 53 452 participants at high risk for lung cancer to either undergo LDCT or CXR, and observed a 20% reduction in lung cancer mortality in the LDCT group.^6^ Long-term follow-up data from the International Early Lung Cancer Action Program (I-ELCAP), which enrolled 31 567 participants in annual LDCT screening between 1992 and 2005, showed that the 80% 10-year lung cancer-specific survival of 484 I-ELCAP participants reported in 2006^7^ persisted after 20 years of follow-up in an expanded cohort of 1257 participants.^8^ The NELSON trial of 13 195 men and 2594 women reported a significant reduction in lung-cancer mortality among those undergoing LDCT screening over an 8- to 10-year follow-up period from randomization.^9^ Importantly, the longest screening interval in NELSON was 2.5 years (between the third and fourth rounds), which subsequent analyses suggested was too long, and the greater mortality benefit observed at years 8 and 9 has been interpreted as consistent with this interval limitation. This observation underpins the use of a shorter screening interval (currently 24 months) in NHS England’s Targeted Lung Health Check program, with ongoing evaluation of whether annual screening should be adopted for higher-risk participants.

### Training and standards

Excellence in radiology reporting is absolutely pivotal to a LCS program because the screening test is fundamentally a radiological one and the accuracy of the LDCT report determines downstream pathway decisions, costs, and patient outcomes. The screening radiologist is, however, only one component of a multidisciplinary screening team. Administrative and call/recall staff, screening practitioners delivering risk assessment, the responsible respiratory physician chairing the screening review meeting (SRM), and wider multidisciplinary team (MDT) colleagues all contribute to the eventual screening decision. Maintaining excellence at the radiology step is therefore necessary but not sufficient. It must be embedded within a multidisciplinary framework with shared accountability for clinical and operational quality.

All NHS England (NHSE) LCS reporting is performed according to the Standard Protocol and Quality Assurance Standards policies.^10^^,^^11^ These documents, which also include a national protocol for the reporting of incidental findings in LCS, form the bedrock upon which the economic case for LCS in England is founded. NHSE mandates that screening readers be registered with the General Medical Council, be a Fellow of the Royal College of Radiologists (RCR), or have completed radiology training and a qualification accepted for equivalence which has led to a Certificate of Eligibility for Specialist Registration. The RCR and British Society of Thoracic Imaging (BSTI) recommend readers report a minimum of 500 thoracic CTs per year in their routine clinical practice (a significant proportion of which should be for the investigation of lung cancer), attend regular thoracic MDT meetings, complete a BSTI/NHSE endorsed CT screening workshop and participate in quality assurance (QA), specifically the need for external quality assurance (EQA) to monitor the ongoing performance of the radiologist. Adherence to these standards is important because reporting screening LDCT in asymptomatic participants is different to reporting clinically indicated CT chest in symptomatic patients.

Beyond minimum case-volume requirements, structured education in LCS is increasingly recognized as essential to the clinical and economic effectiveness of any national screening program.^12^ Internationally, the European Society of Thoracic Imaging (ESTI), endorsed by the European Society of Radiology, has developed the Lung Cancer Screening Certification Project to standardize reader training across Europe, while the International Early Lung Cancer Action Program provides a freely accessible online teaching file. A recent comprehensive review by Shaham and Kazerooni^12^ argues that structured radiologist training, anchored in validated educational tools such as the I-ELCAP Teaching File, the ESTI Certification Project, and the UK’s PERFormance Evaluation for CT Screening (PERFECTS) EQA platform, is fundamental to maintaining diagnostic excellence in LDCT-based screening. In the UK context, structured trial-based experience has informed best practice. The Yorkshire Lung Screening Trial (YLST) demonstrated that protocolized review of a randomly selected 5% of negative scans, alongside review of all subsequent extrapulmonary and interval cancers, is feasible at program scale and provides a template for embedded educational mechanisms such as Radiology Events and Learning Meetings (REALM).^13^ The near real-time QA framework described in the remainder of this article is, by design, primarily educational rather than punitive: its purpose is to surface deviations from expected practice early enough that they can be addressed by feedback and iterative refinement rather than by formal remediation. We view minimum case volumes, accredited workshop attendance, EQA participation, and structured peer-to-peer education within a national reporting network as complementary rather than alternative components of a multi-layered training ecosystem.

### National EQA

To fulfill the requirement for external EQA, the PERFECTS program was developed. PERFECTS is a vendor-neutral, web-based EQA platform that incorporates a Digital Imaging and Communications in Medicine (DICOM) file viewer to display images and incorporates standard measurement and annotation tools. All radiologists reporting LCS in NHSE must participate in PERFECTS annually to comply with national QA standards.^11^ Reporters receive bespoke feedback on their screening performance with national bench-marking to their peers. A key strength of PERFECTS is its capacity to statistically identify under-performing radiologists and provide targeted suggestions for improvement, in a manner directly analogous to the mature PERFORMS scheme used in breast screening. The PERFECTS outlier procedure defines mild and severe outliers according to multiples of the standard deviation from the mean and applies proportionate remedial actions which may include monitoring on the next round of EQA for mild outliers or re-sitting the EQA and discussing performance with the relevant responsible radiologist(s) for severe outliers.^14^ The use of national EQA for other cancer screening, for example in breast, has the potential to predict and identify suboptimal performance in real-life practice.^15^^,^^16^ Importantly, PERFECTS is mandatory and covers every NHSE LCS reader; the framework described in the remainder of this article is therefore intended to complement, not replace, PERFECTS.

### Near real-time QA and routine double reading

The current national EQA tests performance in a curated dataset. Real-life reader performance data are typically considered “gold-standard.” However, it is important to distinguish 2 separate real-life measures: (1) the per-reporter cancer detection rate, which has very low statistical power as a near real-time outlier signal because of the 1%-1.5% per-screen cancer prevalence, and (2) the per-reporter referral rate (lung nodule and incidental findings), which is a much higher-frequency signal and is therefore amenable to near real-time statistical monitoring. The near real-time framework described in this article focuses on the latter; near real-time use of cancer detection rate would require accumulation of data over several years before a statistically meaningful signal could emerge, by which time considerable patient harm could have occurred. Other data may be used as a surrogate marker of radiologist performance in real-time, such as the proportion referred for interim imaging or for an incidental finding. Individual data can be compared with thresholds and if feedback is provided to change practice, reporters may be prevented from becoming an outlier in the first place. The lack of ground truth means that it has to be assumed that aiming to be closer to the average reflects correct decisions. The breadth and depth of LCS reporting experience are important for the individual but also for the pool to which the radiologist is bench-marked against. The HLH Imaging Group (HLH) is uniquely positioned to provide such robust and rapid near real-time QA. This would act as an adjunct to already established EQA. HLH was founded in June 2020 by subspecialist thoracic/cardiothoracic radiologists to connect cardiothoracic radiologists in the United Kingdom to provide their expertise to LCS participants regardless of geography. Via a secure cloud picture archiving system (Cimar UK) with integrated vendor-neutral lung nodule artificial intelligence with a structured template report, HLH has grown to be one of the largest single providers of LCS reporting, having reported >1 million LCS studies by 180 subspeciality reporters since 2020, with >40 000 LCS scans reported each month at the time of writing (Figure 1).

**Figure 1:**
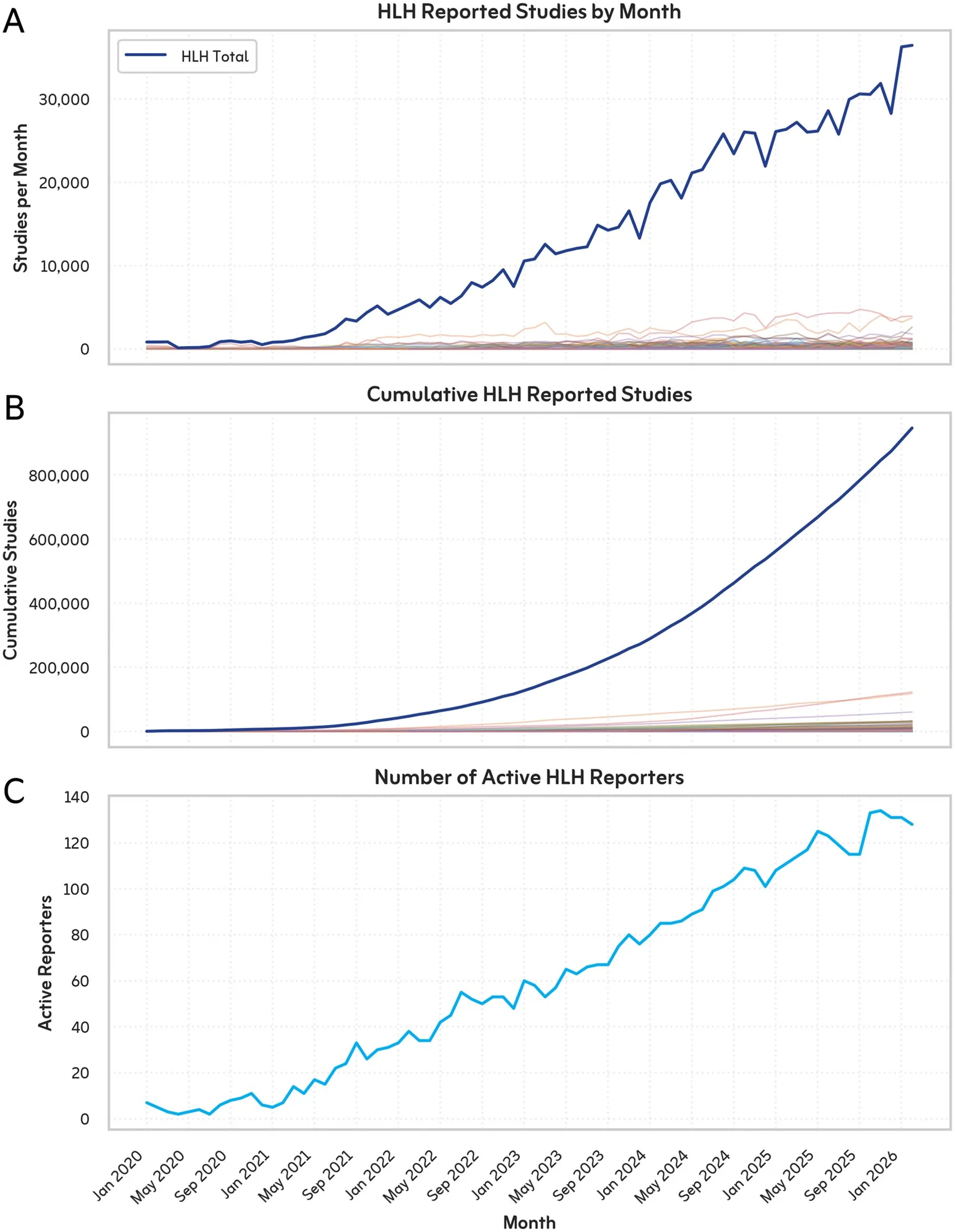
Growth of the HLH Imaging Group lung cancer screening reporting network. (A) Monthly number of low-dose CT (LDCT) lung cancer screening studies reported by HLH and each individual site since program inception. (B) Cumulative number of studies reported by HLH and for individual sites, approaching 1 million examinations. (C) Number of active HLH reporters over time. Together these illustrate the rapid expansion and scale of the HLH reporting network.

### Recall rates

Every quarter, HLH’s data and analytics division calculate the following recall rates for all sites and all reporters (Table 1). Referral rates to the SRM are calculated for different metrics (Figure 2). Active near real-time outlier tracking is performed relative to the thresholds set by NHSE, and relative to statistically-derived bounds from the contemporary reporter pool (see “Addressing outliers” section below). Outlier tracking is performed across global reporting LCS practice rather than per reporter per site to ensure the most holistic reporting assessment (Figure 3). A minimum reporting volume of 125 LCS reports per reporter per quarter is required to equate to a minimum of 500 LCS reports per annum to ensure reporters who are just starting to report LCS are not unfairly penalized by percentage-based thresholds. If a reporter has a referral rate in excess of NHSE’s threshold in any metric in a quarter, they are notified and actively monitored. HLH also provide reporters with a granular break-down of their reporting performance relative to the pool of HLH experts. The bespoke, individualized performance data are intended to serve as an early warning system that enables reporters to reflect on their current reporting practice and referring thresholds and iteratively adjust their reporting. This may help a reporter who is above an NHSE threshold in 1 quarter return to acceptable recall rates in the next or it may mitigate a reporter who is approaching the threshold in 1 quarter from formally triggering on the next quarter. In this regard, the near real-time analysis and synthesis of reporting data in an easily digestible format facilitates the QA process transitioning from a pure outlier-detection process to a continually refining educational endeavor. To ensure complete transparency, each quarter sites receive tracking data on: 1) reporters who have newly breached a referral threshold, 2) reporters who previously breached a referral threshold but have now returned to below threshold, and 3) reporters who have breached a referral threshold for 2 consecutive quarters (Figure 4). If a reporter has a referral rate which exceeds NHSE’s threshold for the same metric in 2 consecutive quarters, they are defined as an outlier and activate HLH’s outlier standard operating procedure (see “Addressing outliers” section below).

**Figure 2:**
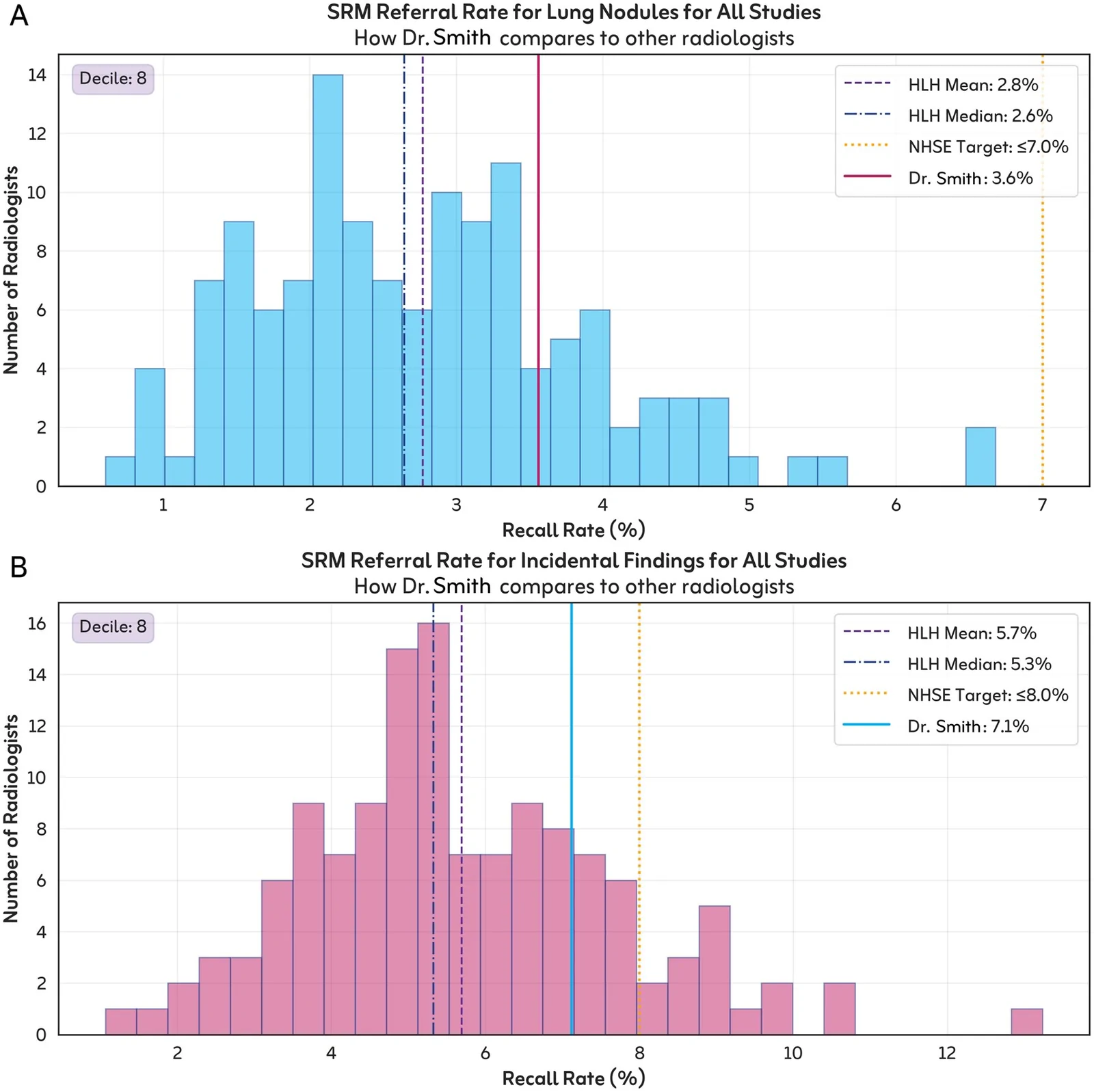
Bar charts of SRM referral rates for A) lung nodules and B) actionable incidental findings for all HLH reporters. Similar plots are generated for HLH sites, with the mean, median and NHSE threshold plotted for comparison. Individualized plots are provided to each site and radiologist to demonstrate how they fit into the HLH distribution which offer the opportunity for analysis and reflection.

**Figure 3:**
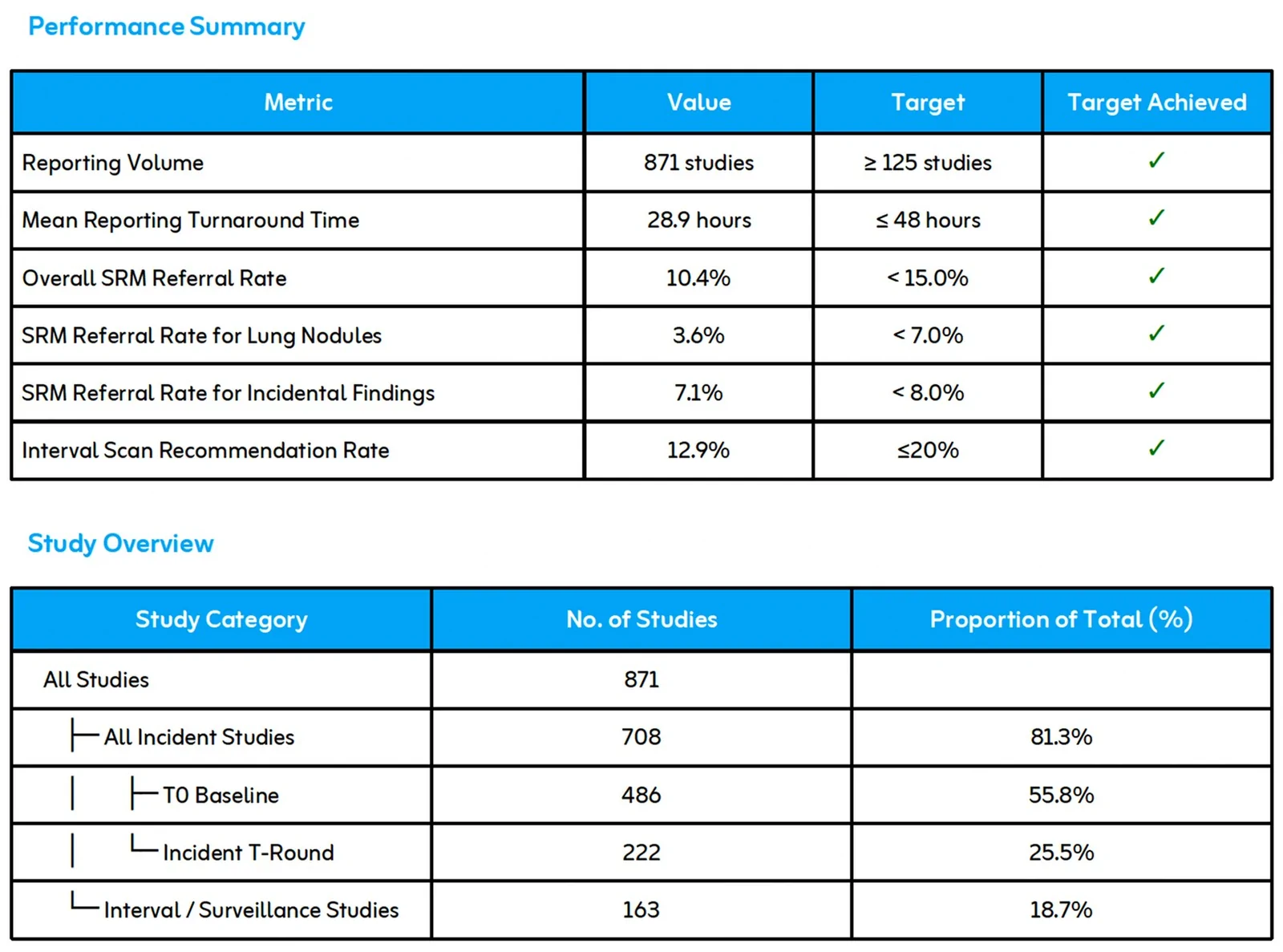
Example summary data from a radiologist’s quarterly quality assurance report. A similar summary is provided to the sites. Incident T-rounds are the LDCT performed at 24-month intervals in the NHSE LCS protocol where no actionable lung nodules are identified. Interval/surveillance studies are the LDCT that occur in between incident T-rounds where actionable nodules are identified and need closer surveillance as per BTS 2015 nodule guidance/NHSE LCS protocol to determine rate of change. Overall SRM referral rate is less than sum of lung nodule SRM referral rate and incidental finding referral rate as some participants are referred for both.

**Figure 4:**
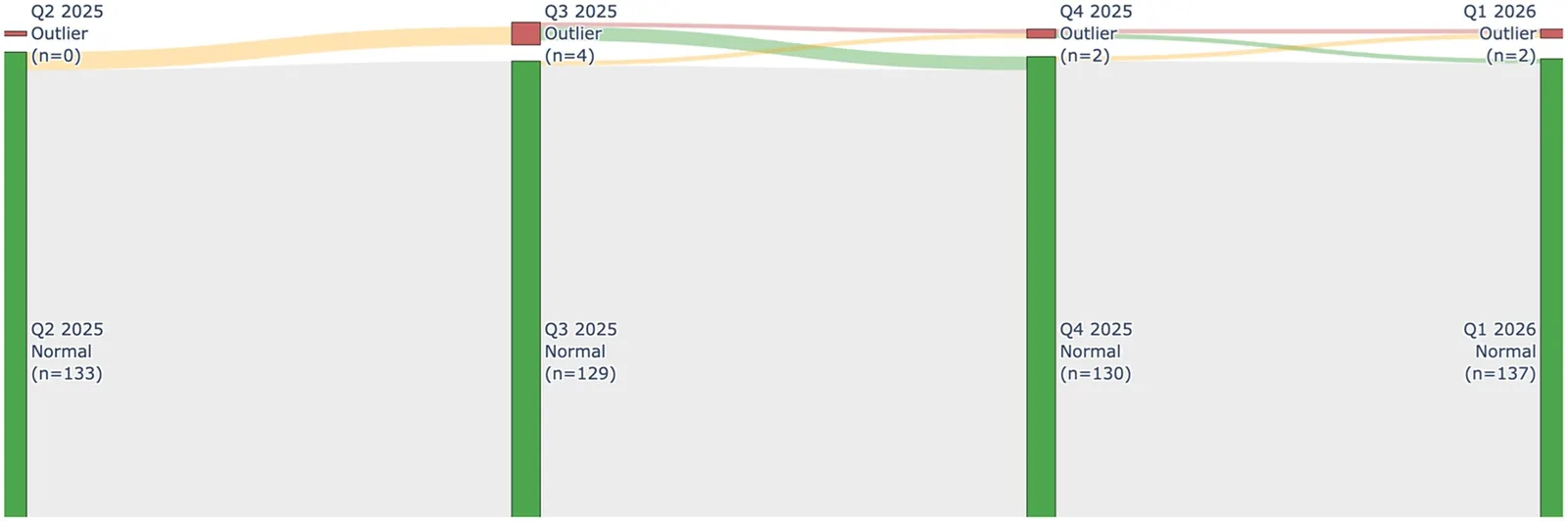
Sankey diagram illustrating changes in reporters’ overall SRM referral rate performance across 2025-26 quarters, where the NHSE threshold is <15%. Between quarters, radiologists returning to expected performance levels transition from red with green line to green, reporters newly breaching the threshold transition from green with yellow line to red and reporters who remain above threshold and become an outlier move from red via line to red.

**Table 1:** A table summarizing the reporting metrics and targets set by NHSE lung cancer screening team.

Metric	Denominator	Target
SRM referral rate	All scans	<15%
SRM referral rate—lung nodules	All scans	<7%
SRM referral rate—incidental findings	All scans	<8%
Interval scan rate for indeterminate nodules	All T-round scans	11%-20%

### Routine double read

The near real-time assessment of reporter recall rates will detect reporters with above average recall rates, with the assumption being that some of these will be false-positive recalls. There is a need to gather near real-time data on potential false negatives. HLH performs routine double reading of a subset of LCS studies to achieve this. Every LCS scan reported by HLH is coded by a proprietary Red-Amber-Green (RAG) diagnostic support triage tool. A LCS scan with no actionable nodule(s) or actionable incidental findings is assigned a “green” RAG rating and deferred to the next screening round, which is currently every 24 months according to NHSE LCS protocol. HLH randomly harvest 5% of negative (green RAG) reports across all incident T-rounds (T0, T1, T2, etc.), stratified by site and by reporter to ensure a balanced and representative dataset. The 5% sampling fraction was chosen to match the random-sampling fraction used in the published Yorkshire Lung Screening Trial (YLST) QA process,^13^ the principal UK precedent for embedded LCS QA, and represents a proportion that does not materially extend reporter workload at program scale. This ensures a subset of cases that will not be routinely reviewed again for 24 months within the LCS program are audited. The cases are randomly assigned to different reporters to double read within bespoke HLH QA software. All second reads are performed by all active HLH cardiothoracic radiology subspecialists, each of whom holds CCT (or equivalent) in clinical radiology, is a Fellow of the Royal College of Radiologists (or has an accepted equivalent qualification), meets the BSTI/NHSE volume criteria of ≥500 thoracic CT per year, has completed the BSTI/NHSE-endorsed LCS workshop, and participates in PERFECTS EQA. Second readers review the images, the original report, and the original RAG triage assignment and record their QA opinion. One of the strengths of our process is that all HLH reporters participate in routine double read, so the QA opinion is that of routine LCS reporters and all reporters have the benefit of seeing many other reporters’ reporting styles and thresholds for action (Figure 5). Cases are graded in accordance with prior published LCS-specific grading^13^:

**Figure 5:**
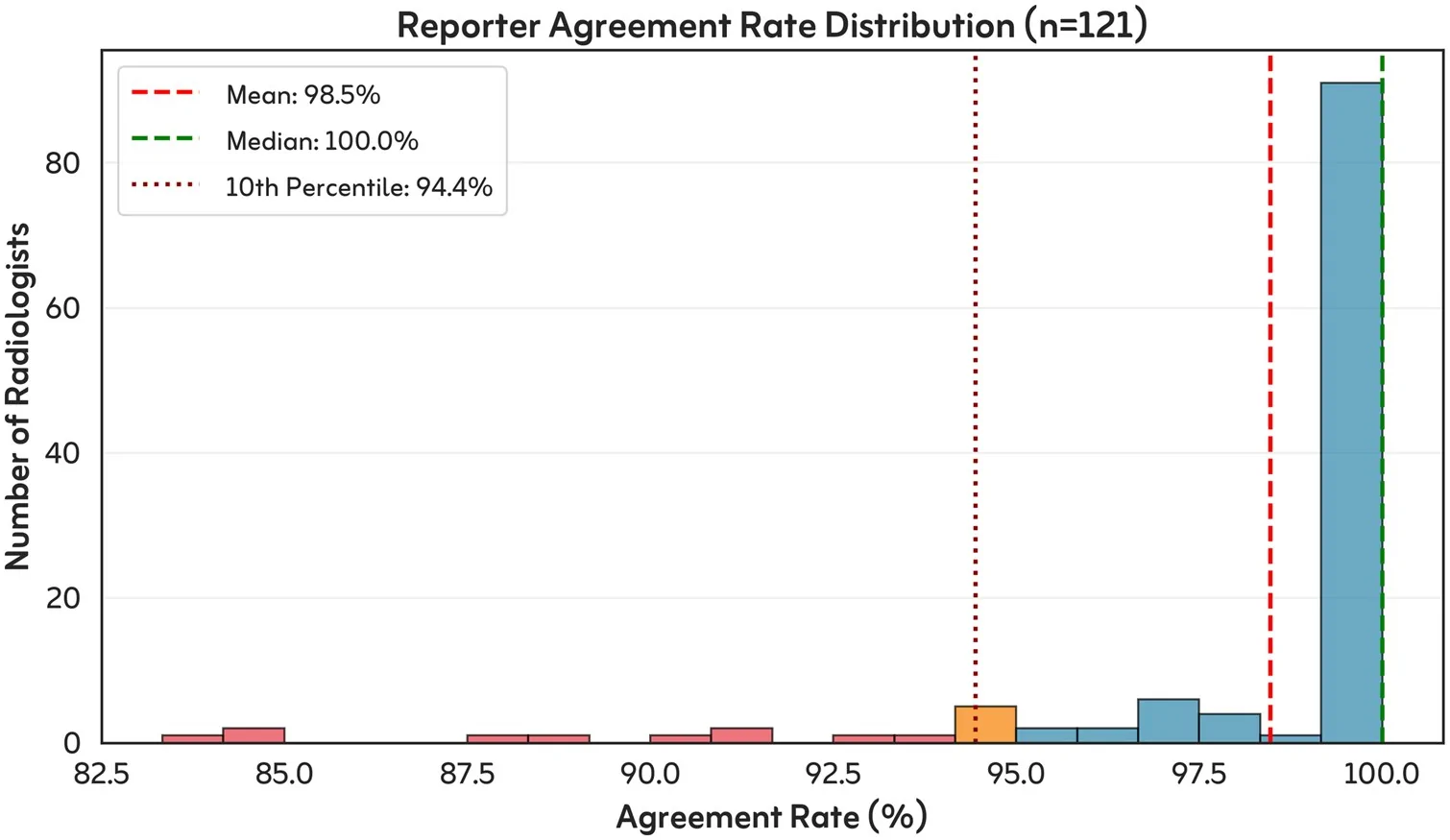
Distribution of reporter agreement rates in double-read quality assurance. Histogram showing the distribution of agreement rates between primary and secondary reads across HLH reporters (*n* = 121). Dashed lines indicate the mean and median agreement rates, with the dotted line marking the 10th percentile of reporter performance.

- Satisfactory
- Satisfactory with learning points
- Unsatisfactory

When a case is graded either as “satisfactory with learning points” or “unsatisfactory” by the initial QA radiologist, the case is then routed to an independent discrepancy panel who are all blinded to initial QA report as well as the other discrepancy panel members’ opinions. Discrepancy panel members are LCS reporters with significant clinical experience having reported LCS for at least 4 years. Consensus grading is performed and if the cases remain clinically actionable the site is informed. The initial reporter is informed on a case-by-case basis in real-time for personal reflection and a summary of all double read outcomes is provided in the QA data packs to each reporter and each site (Figure 6).

**Figure 6:**
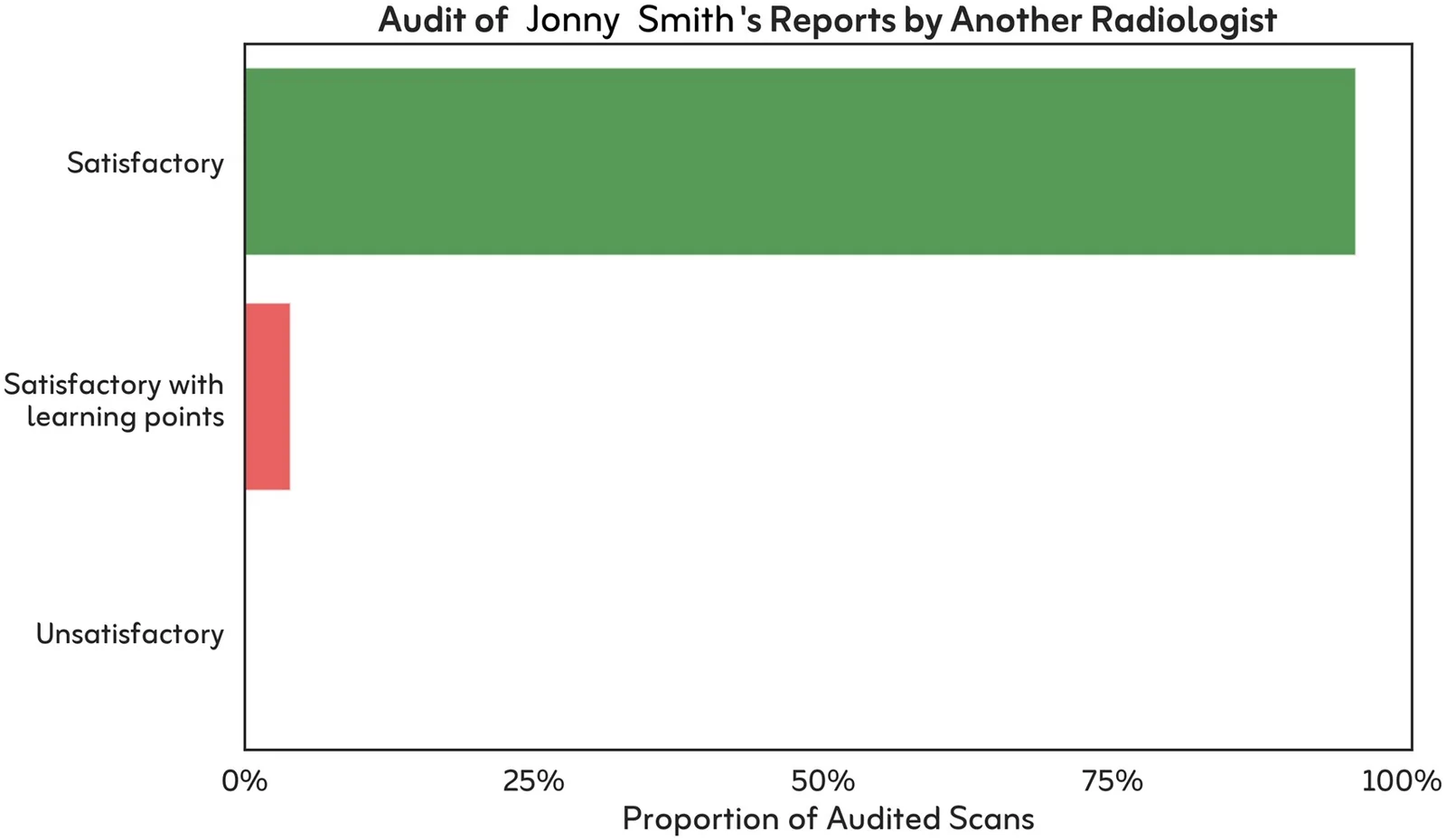
Outcomes of routine double-read quality assurance for a single radiologist or site. Proportion of audited cases classified as satisfactory (normal study or minor issues without harm), satisfactory with learning points (moderate issues of clinical significance), or major error following independent second reading and discrepancy review. The majority of cases were graded satisfactory, with a small proportion requiring learning points and very few major errors.

### Addressing outliers

The NHSE Quality Assurance Standards^11^ clearly define expected performance metrics across a range of domains, on both a quarterly and annual basis. With regards to radiologist performance the relevant thresholds are summarized in Table 1. The Standards outline that “*100% of outliers, as defined from a quarterly or annual review, will have evidence of agreed actions (including a period of double reporting) with the responsible radiologists.*” and “*Once outliers are identified, feedback can be provided to responsible and individual radiologists and SRMs for education and calibration*.”^11^ However, the Standards do not stipulate (1) the minimum sample of cases that should be retrospectively reviewed once outlier status is triggered, (2) the criteria (in terms of concordance rate or absolute change in referral rate) for returning a reporter to routine reporting, (3) the threshold of discrepancy at which formal supervised reporting or further training is mandated, or (4) the time-frame over which sustained out-of-range performance constitutes a persistent rather than a transient signal.

#### A simple statistical framework for outlier definition

Defining what constitutes a meaningful breach of a threshold benefits from a simple statistical framework. A single quarterly observation of a reporter’s referral rate is subject to binomial sampling noise: at the minimum quarterly volume of 125 reports and a true underlying referral rate of around 5%, the standard error of the observed rate is approximately √(*p*(1−*p*)/*n*) = √(0.05 × 0.95/125) ≈ 1.9%. A single-quarter excursion of approximately ±2% around the true rate is therefore expected by chance alone. Requiring that the threshold be exceeded in 2 consecutive quarters before triggering formal outlier status substantially reduces the probability of false-positive outlier designation: assuming independent quarters and a per-quarter false-positive probability of .05, the joint probability is approximately .0025. This is conceptually analogous to (though much simpler than) the multi-SD outlier procedure used in PERFECTS, where mild and severe outliers are defined according to standardized distances from the mean.

This approach can be combined with, or used in place of, fixed thresholds. We analyzed empirical referral data for all HLH reporters meeting the minimum reporting volume across the 2025-26 financial year (*n* = 136 reporters; Figure 7). For lung-nodule referrals the empirical distribution had mean 2.74% (SD 0.85; median 2.62%, MAD 0.77); the parametric mean + 2SD upper bound was 4.44% and the robust median + *k*·MAD bound (*k* = 1.4826, the Gaussian consistency factor) was 4.54%. For incidental-finding referrals the empirical distribution had mean 5.71% (SD 1.77; median 5.80%, MAD 1.82); the corresponding upper bounds were 9.25% and 10.36%. The close agreement between the parametric and robust estimators supports their use in this dataset. A sensitivity analysis of the number of reporters flagged at different candidate thresholds is shown in Figure 8. At the current NHSE lung-nodule upper threshold of 7.0%, no reporter would be flagged in 2025-26; at the mean + 2SD threshold (4.5%) 5 reporters would be flagged. Conversely, for actionable incidental findings, the NHSE threshold of 8.0% flags 10 reporters, while a pragmatic statistically-derived threshold of 9.0% would flag 6. In the contemporary HLH reporter pool the empirical mean + 2SD upper bound for lung-nodule referral therefore lies well below the NHSE fixed threshold (4.44% vs. 7.0%), whereas for incidental findings the 2 bounds are closely matched (9.25% vs. 8.0%). This suggests that the current fixed lung-nodule threshold may be insufficiently sensitive in the modern program and may merit re-calibration against contemporary reporting data, while the incidental-findings threshold is broadly consistent with the statistically-derived bound. Importantly, our data also demonstrate that lower thresholds are feasible. A proposed lower threshold of 1.0% for lung-nodule referrals (close to the mean − 2SD of 1.04%) flagged a single reporter, as did a proposed lower threshold of 2.0% for incidental-finding referrals (close to the mean − 2SD of 2.17%). This offers a means of identifying potential under-callers in near real-time. We acknowledge that statistically-derived thresholds drift with the underlying reporter pool, and that lower thresholds in particular need calibration against confirmed cancer detection outcomes before being used punitively. As such, they are most appropriate as educational triggers rather than as formal remediation triggers.

**Figure 7:**
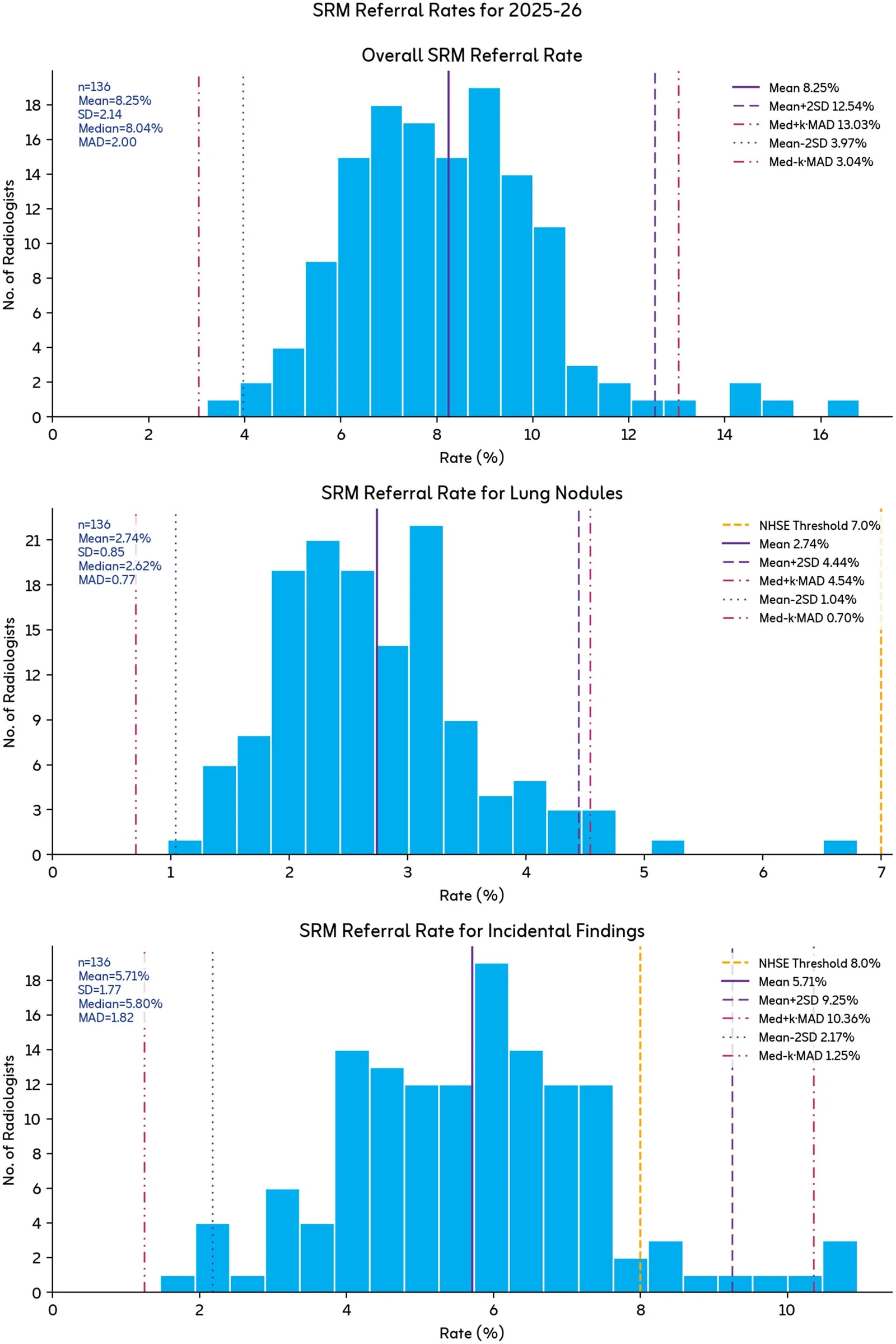
SRM referral-rate distributions for HLH reporters across the 2025-26 financial year (*n* = 136 reporters). The 3 panels show the empirical distributions of overall SRM referral rate (top), lung-nodule referral rate (middle), and incidental-finding referral rate (bottom). Vertical reference lines indicate the mean, parametric statistical thresholds (mean ± 2SD), robust statistical thresholds (median ± *k*·MAD, where *k* = 1.4826 is the consistency factor that recovers 2SD under Gaussian assumptions), and where applicable the current NHSE’s fixed threshold. The close agreement between parametric and robust thresholds supports the suitability of statistically-derived bounds in this dataset.

**Figure 8:**
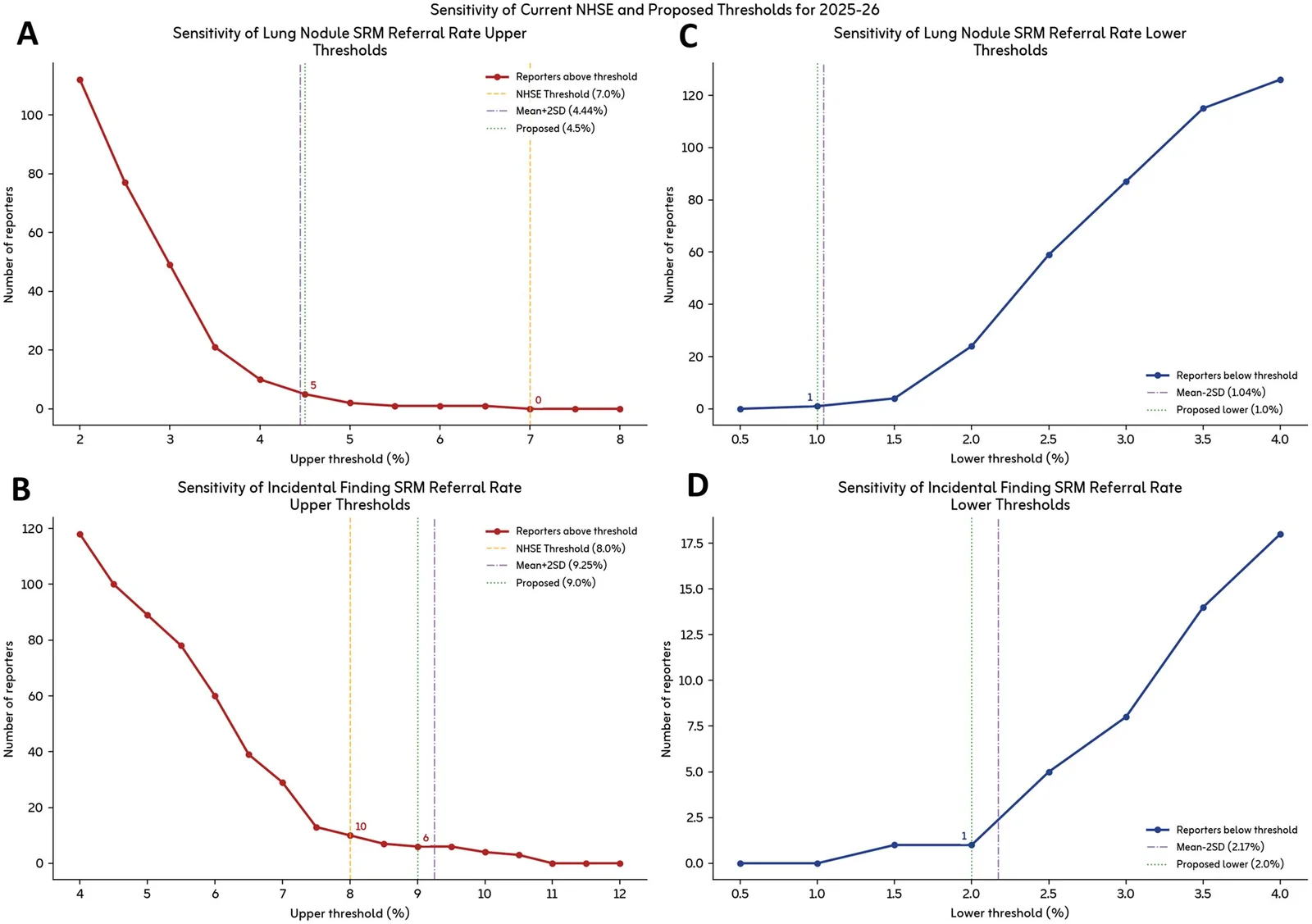
Sensitivity of upper and lower thresholds for lung-nodule and incidental-finding SRM referral rates in 2025-26. (A) The number of reporters flagged as exceeding a given upper threshold for the lung nodule referral rate to the SRM. (B) The number of reporters flagged as exceeding a given upper threshold for incidental findings referral rate to the SRM. (C) The number of reporters who would fall below a lower threshold for lung nodule referral rate to the SRM. (D) The number of reporters who would fall below a lower threshold for incidental findings referral rate to the SRM. Vertical reference lines indicate the current NHS England threshold (orange dotted line where applicable), the mean ± 2SD statistical threshold (purple dotted and dashed line), and a pragmatically rounded “proposed” threshold (green dotted line). Annotated numbers indicate the reporter count flagged at the NHSE threshold (adjacent to orange dotted line) and at the proposed thresholds (adjacent to green dotted line). These data show that statistically-derived thresholds materially alter the number of reporters flagged and that defining a lower threshold is feasible without flagging large numbers of reporters with naturally lower referral rates.

#### Operationalization in HLH

Following real-time review of data it became evident that a radiologist may exceed the threshold for referral rate (particularly for incidental findings) due to factors such as case mix, impact of site-related socioeconomic and demographic variance, and clustering. As such the radiologist may be reporting “correctly” and entirely to protocol^11^ but still inadvertently exceed the threshold. Ultimately the aim of the outlier process is to detect and improve performance outside expected limits, and to maximize the benefits of the LCS program. Initial experience of reviewing “outliers” found that a radiologist may exceed the referral threshold for 1 quarter (often only very marginally) and then in the subsequent quarter return to “accepted” levels of practice, particularly following targeted feedback to refine performance—a pattern consistent with the binomial noise argument above. On both statistical and operational grounds therefore, we regard any radiologist who exceeds an NHSE metric for 2 consecutive quarters as being an outlier and thus requiring formal action (Figure 9).

**Figure 9:**
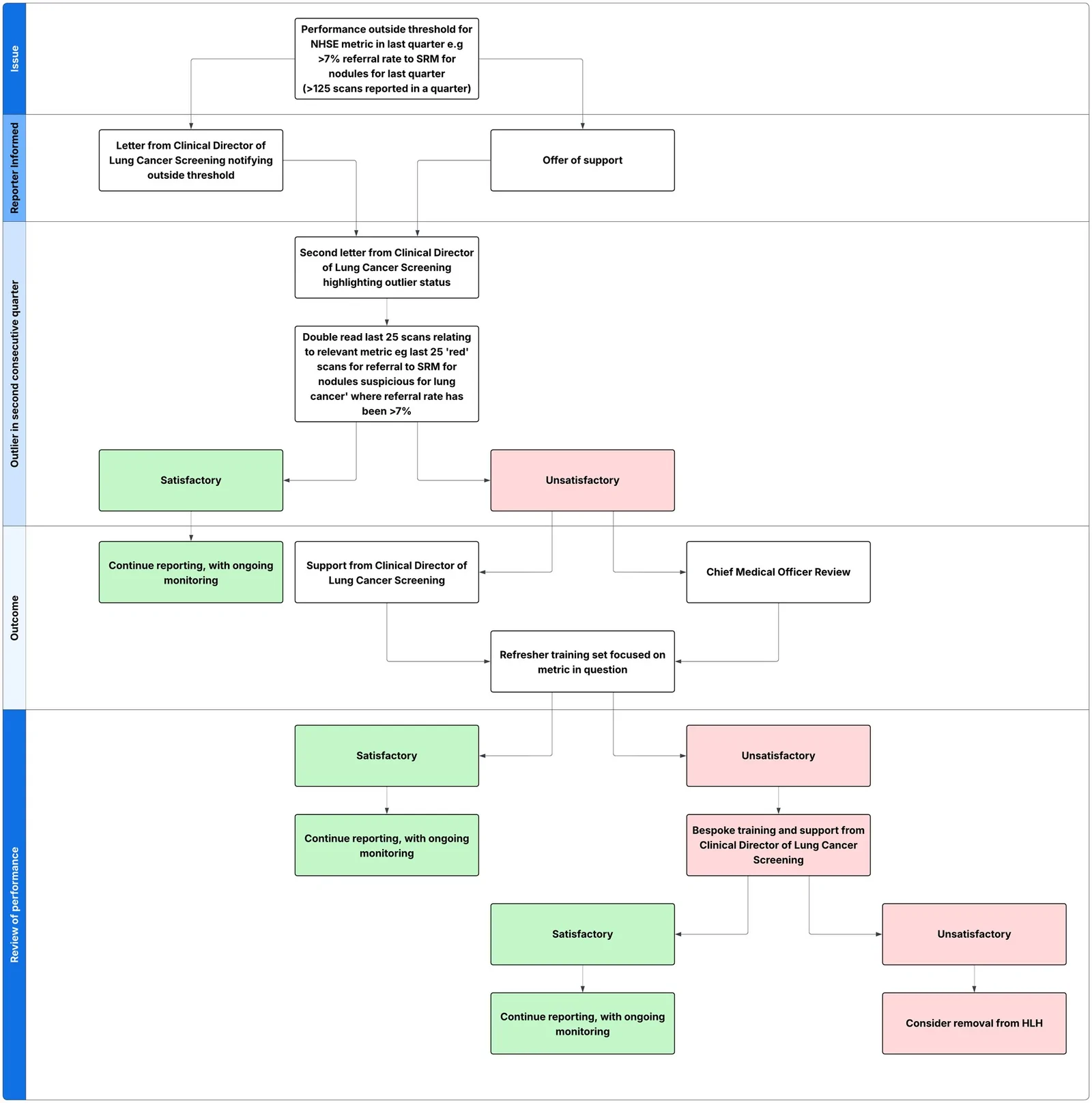
Flow chart demonstrating HLH’s approach to address outliers in lung cancer screening.

If a radiologist is identified as having exceeded the threshold in a relevant domain, they receive granular data regarding the nature of their performance and are informed they have exceeded the threshold; for incidental findings this includes a specific breakdown by type. If they exceed the referral threshold on a second consecutive quarter they receive formal notification of outlier status in that domain from the HLH clinical leadership team. Given the subjective nature of many referral decisions, the most important factor is to ensure that the finding truly warrants further action in the context of the LCS program. As such, in keeping with national guidance, a period of targeted double reading by a radiologist with subspecialist expertise in LCS is undertaken. Given a large number of LCS scans are “normal” (i.e. without significant findings), and the assignment of outlier status is specifically based on exceeding parameters (i.e. aiming to identify “overcallers”), double reading is specifically targeted to the characteristic that has been exceeded. For example, if a radiologist is an outlier in terms of incidental findings referral rate to SRM, a retrospective review of the last 25 cases referred for actionable incidental findings will be double read for concordance. The figure of 25 was chosen to balance the need for a sample large enough to yield meaningful concordance data (25 cases gives an exact binomial 95% confidence interval of approximately ±18% around an observed 80% concordance rate) with the workload imposed on expert double readers, and corresponds to approximately 1 to 2 months of actionable incidental findings referrals at typical volumes. The results of this double reading process are summarized by the HLH clinical leadership team and fed back to the radiologist, along with bespoke advice and guidance to refine practice. Reassuringly radiologists have tended to revert to normal levels following this intervention. If outlier status were to persist across multiple subsequent quarters further training and support would be provided, tailored to the radiologist’s practice. This could include a period of supervised reporting if required. A similar process of focussed double reading is undertaken if a radiologist exceeds expected values in a domain; e.g. an interval CT recommendation rate >20% would trigger double reading of the last 25 scans where an interval scan was recommended (“orange”). The real-life dynamic impact of this process between quarters is illustrated in Figure 4.

## HLH lessons learned reporting LCS for >5 years in one of the largest LCS programs

There are numerous lessons to be learned regarding quality assurance and standards as an LCS program matures within the real-world healthcare setting. With over 1 million LCS scans reported, HLH has garnered extensive experience of both reporting and monitoring of near real-time quality assurance, as well as outlier management; we share these reflections as a provider’s experience and acknowledge that the framework described will need to be tested in other UK reporting networks and internationally before generalizable conclusions can be drawn. In addition to bench-marking radiology reporters against the LCS nodule and incidental finding recall rate thresholds set by NHSE, we have learned that providing each reporter with bespoke granular data on their reporting appears to support reflection on individual reporting practice, though we do not yet have formal survey data on reporter perception.

Each quarter, reporters are provided with data comparing their individual recall rates for each nodule subtype (Figure 10) and each actionable incidental finding subtype against HLH mean (Figure 11). Although we cannot at present verify, by formal survey, the extent to which each reporter engages with this material, the observed convergence of new reporter referral rates with the pooled HLH mean over time (Figure 12), and the return-to-threshold pattern observed in the small number of reporters who have approached or exceeded an NHSE threshold (Figure 4), is consistent with reporters engaging with and acting upon their bespoke feedback. This approach is intended to leverage the scale of HLH’s collective LCS experience across what is a large UK cardiothoracic radiologist reporting network. This observation is consistent with the wider literature on structured education in screening, in which integration into a reporting network with embedded feedback shortens the learning curve for newer reporters.^12^ They also have the scaffolding of a subspecialty radiology network through which effective training and mentoring can occur in real-time on a daily basis.

**Figure 10:**
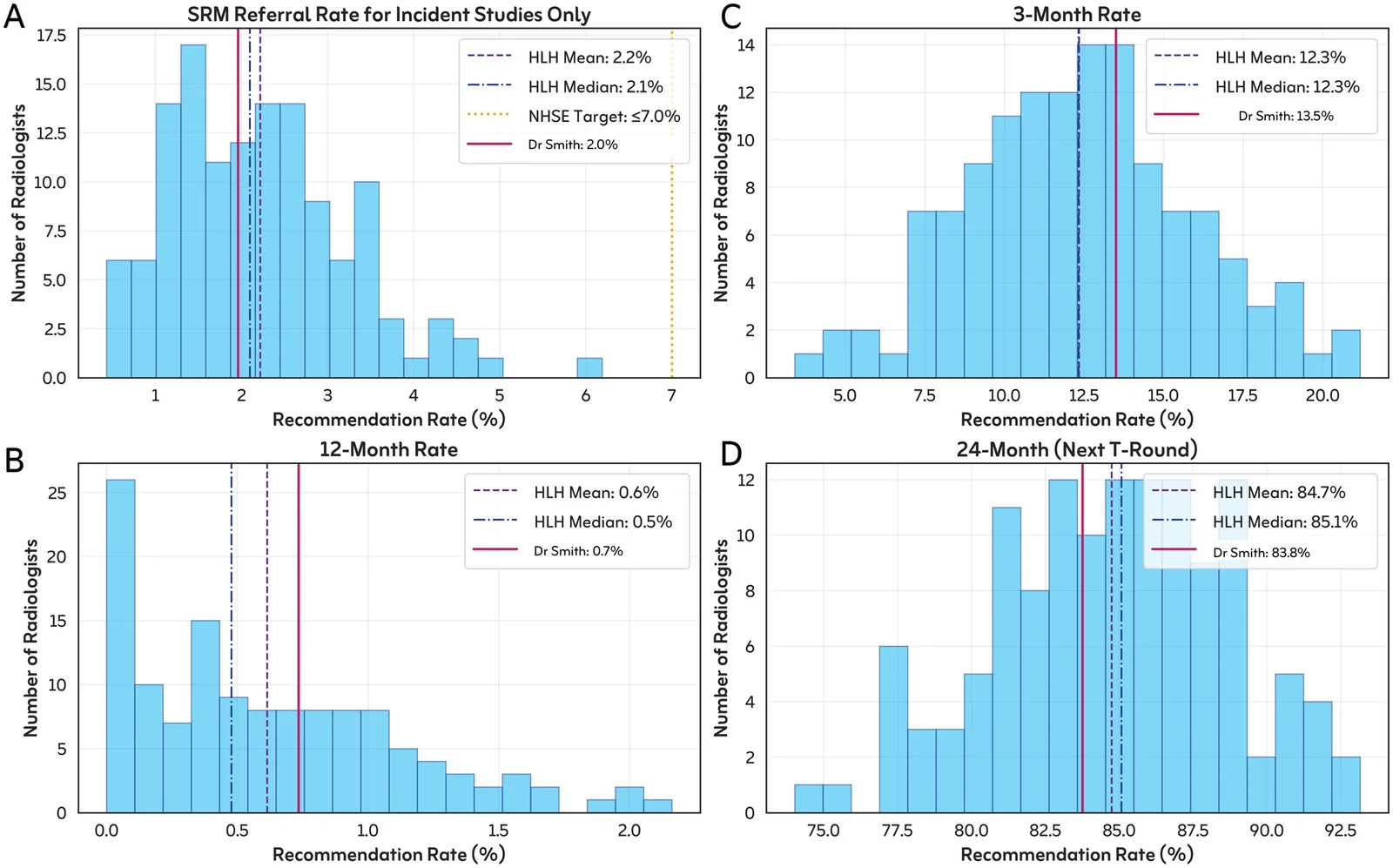
Distribution of SRM referral and surveillance recommendations across HLH reporters. Histograms show the distribution of recommendation rates across the HLH reporting pool for (A) SRM referral for incidental findings, (B) 12-month follow-up, (C) 3-month follow-up, and (D) routine 24-month (next T-round) screening. Dashed lines indicate the HLH mean and median, while the solid red line indicates the individual reporter’s rate, enabling comparison with peers and with national thresholds where applicable.

**Figure 11:**
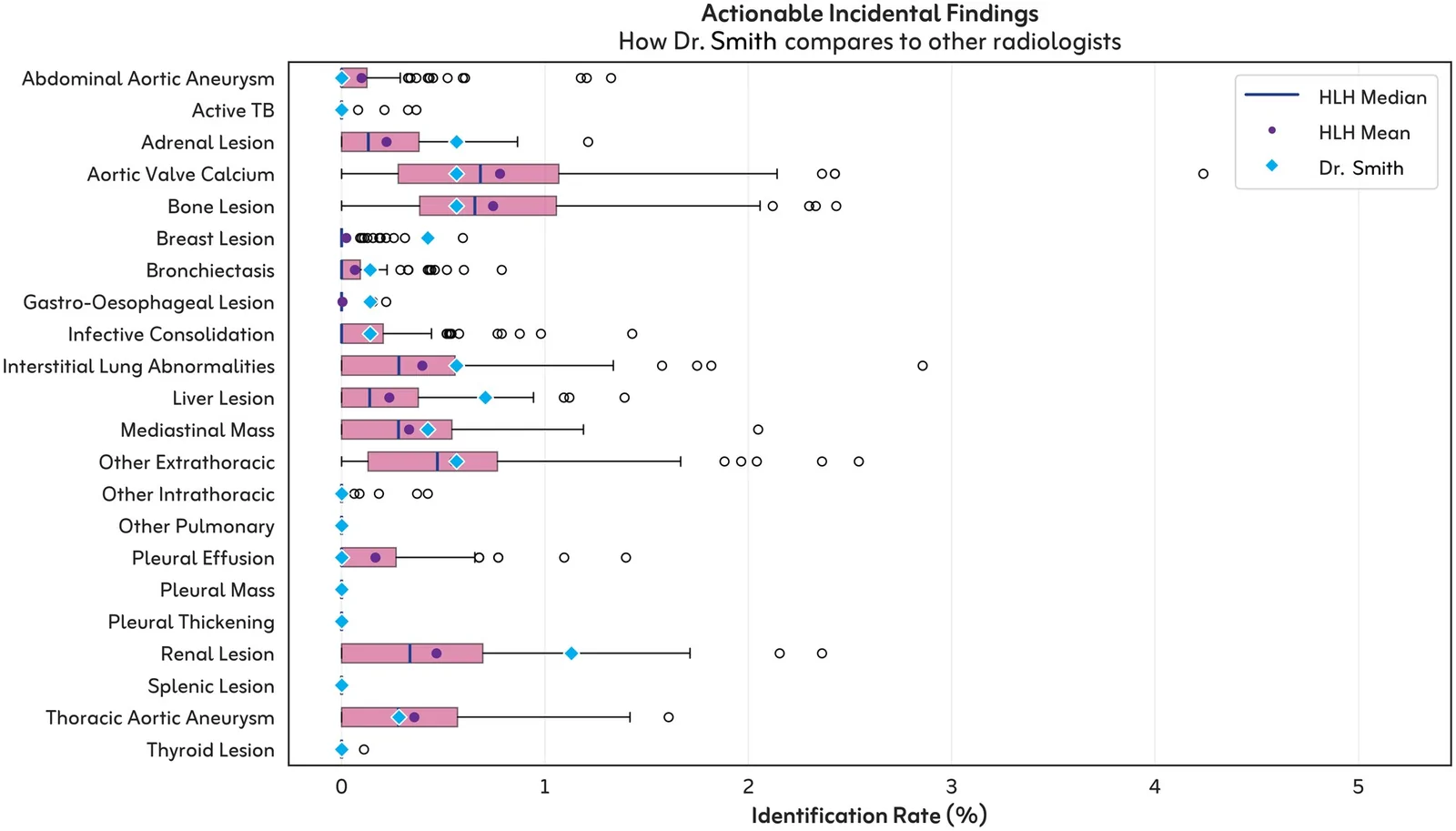
Example of granular reporter feedback for actionable incidental findings. Box-and-whisker plots show the distribution of identification rates for each incidental finding category across HLH reporters. The pink boxes represent the interquartile range, with the median shown as a dark blue line and the mean as a purple circle. Whiskers indicate the typical range (1.5× IQR), and open circles denote outlying values. The sky-blue diamond indicates the individual reporter’s rate, allowing comparison with peers across each finding category.

**Figure 12:**
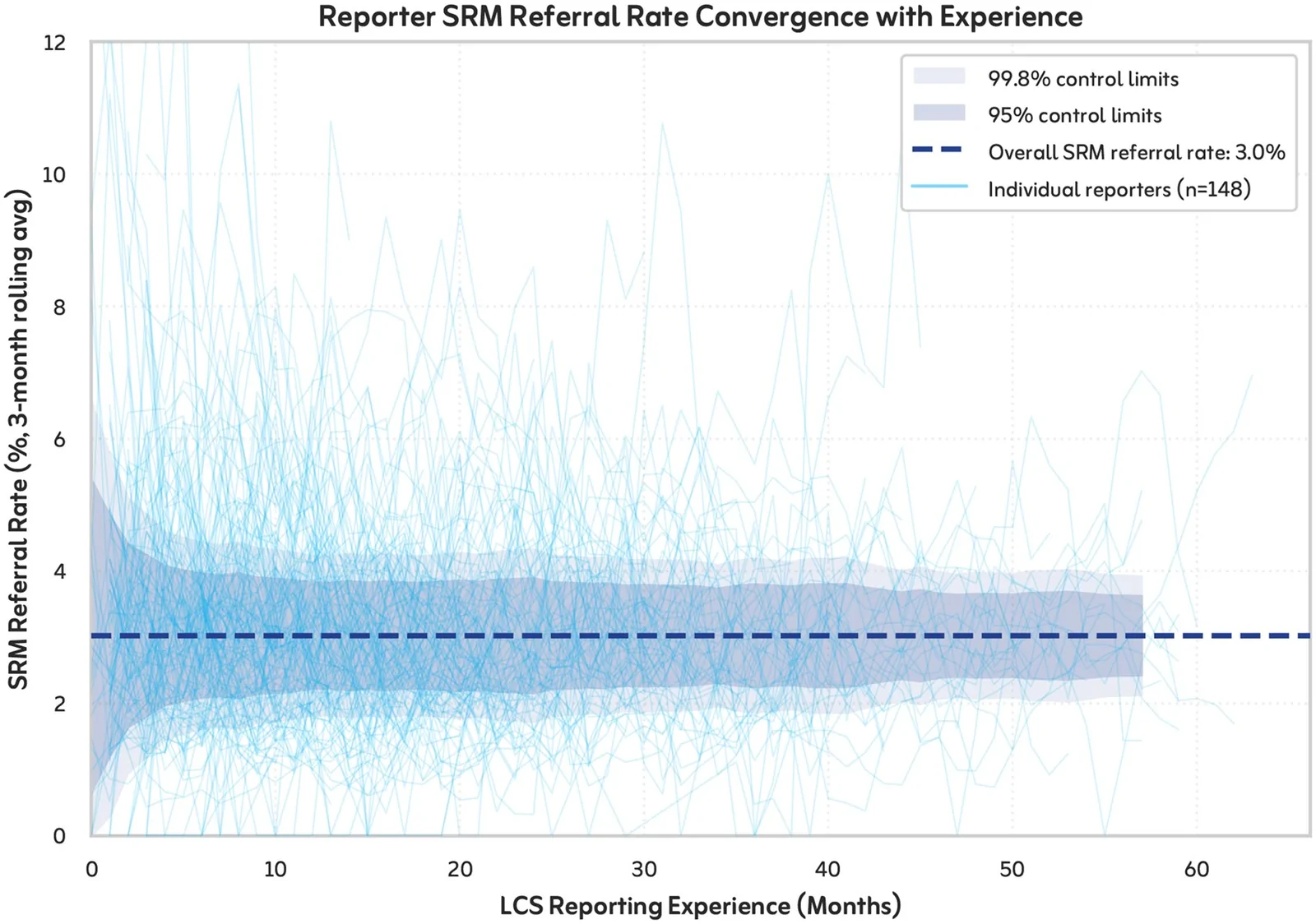
Convergence of reporter SRM referral rates with cumulative reporting experience. Each line represents the rolling-average SRM referral rate of an individual HLH reporter (*n* = 144), aligned to their first month of lung cancer screening (LCS) reporting. The dashed line shows the pooled SRM referral rate (3.0%), below the NHS England threshold of 7.0%. Early variability reflects low reporting volumes. However, rates stabilize and converge with increasing experience, consistent with effective near real-time quality assurance and feedback.

### Future directions

Current NHSE focus is on identifying reporters with nodule and incidental findings rates above their thresholds. This is clearly important to ensure the program remains clinically and cost-effective as it scales toward national rollout. However, in the future, attention should also be devoted to defining an acceptable lower limit of nodule and incidental recall rates. While both reporter sensitivity and specificity are tested in PERFECTS EQA, near real-time QA data to potentially identify under-callers in LCS would be prudent. As discussed above, this proposal applies to per-reporter referral rate (not cancer detection rate); referral rate is high-frequency enough to support near real-time monitoring, whereas cancer detection rate would require accumulation over several years before a statistically meaningful signal could emerge. The empirical 2025-26 data presented in Figures 7 and 8 demonstrate that statistically-derived lower thresholds (mean − 2SD or equivalent) are tractable in a large reporting network, identifying only a small number of reporters at the extreme of the distribution and thus providing a high-specificity early-warning signal without unduly penalizing reporters whose practice is naturally at the lower end of the normal range. Confirmation that low referral rates correspond to true under-detection of cancer would require linkage to interval-cancer outcomes and is therefore consistent with a time-frame of years rather than months.

#### SRM concordance as an additional near real-time metric

A natural extension of the framework presented here is to benchmark each reporter’s SRM referrals against the SRM panel’s outcome decisions. The SRM is typically a multidisciplinary expert panel and will, by design, disagree with a proportion of reporter referrals. The reporter-level proportion of SRM referrals validated by the SRM as warranting further work-up provides a near real-time measure of referral specificity which is conceptually distinct from the absolute referral rate. We are currently developing the data linkage required to report this metric back to reporters in the quarterly QA data pack, and we believe SRM concordance should be considered for inclusion in any national near real-time QA framework. However, currently, there is no formal QA or EQA of SRM practice, and local pathway decisions (such as if/when to biopsy and threshold to action certain incidental findings) may vary significantly across the national landscape which also needs to be addressed in due course as the LCS program expands.

#### Relationship to the NHSE effectiveness standards

The NHSE QA Standards (addressed in the body of this article) operate at the reporter-level. They are complemented by the NHSE Effectiveness Standards, which operate at the program-level and concern outcomes such as cancer detection rate, stage shift, uptake and interval-cancer rate. The near real-time data infrastructure described here directly supports those Effectiveness Standards whose denominator is within the imaging dataset, e.g. the proportion of scans triggering SRM referral, the proportion of incidental findings by category, the distribution of nodule sizes and types, the proportion of scans recommended for early interval imaging, and the proportion of scans recommended for return to routine screening. Several of the current Effectiveness Standards have imaging-only denominators and could in principle be reported back to reporters and sites in near real-time within this framework. Standards that depend on linkage to cancer-registry or pathology data (cancer detection rate, stage at diagnosis, interval-cancer rate, overall and lung-cancer-specific mortality) can only be reported annually and require external data flows. Aligning the near real-time framework with the Effectiveness Standards is, in our view, a priority for any national standardisation effort.

A key remaining question is whether outlying values in these metrics identify radiologists who are more likely to miss opportunities for early detection of lung cancer. In breast cancer screening, individual scores from the UK PERFORMS national test-set-based self-assessment scheme for mammography have been shown to act as a useful surrogate for real-world screening proficiency, particularly for detecting underperforming readers.^15^^,^^16^ Comparable validation studies are now required in the context of lung cancer screening; the existing PERFECTS infrastructure, combined with near real-time referral-rate data of the kind presented here, provides the foundation on which such studies can be built.^14^

Addressing error and discrepancies within radiology is a complex topic which has been addressed by many excellent reviews and also reviewed by the RCR.^17^ Even within the setting of routine clinical reporting, we do not have an agreed metric as to what level of discrepancy/error is acceptable for an individual radiologist. Large reviews and commentaries report that in routine daily practice radiologists have real time interpretive errors in about 3%-5% of examinations with higher error rates on retrospective reviews up to 30%.^18^ In the prespecified NELSON analysis of screening performance and interval cancers, 35% of interval cancers were retrospectively not visible on the prior CT, but 50% were visible and were classified as due to radiological detection/interpretation errors (with the remainder due to protocol misclassification, noncompliance, etc.). A QA process described within the YLST^13^ reported that only 1% of randomly selected negative scans were ultimately judged “unsatisfactory” and proposes this blinded, graded review plus Radiology Events and Learning Meetings (REALM) as a template QA model for national programs. In population-based breast screening programs, mammography remains the primary modality for identifying breast malignancy. Nevertheless, evidence from several trials indicates that diagnostic inaccuracies persist, with studies reporting error rates up to 30% in screening interpretations.^19^^,^^20^ We believe there is a need for the introduction of a holistic reporter review metric that brings together multiple quality indicators including double-read outcomes, incidental discrepancies, and PERFECTS EQA to track global LCS performance. Such an integrated approach would enable more accurate monitoring of real-world performance, support early detection of underperformance, and enhance quality assurance processes. In turn, it offers an opportunity to identify emerging issues at an early stage and to guide targeted training and professional development initiatives. The implementation of an LCS program is inherently associated with a period of adaptation for radiologists as they develop experience within this specific clinical context. Systematic analysis of discrepancy databases from LCS programs may enable the identification of recurrent and characteristic patterns of interpretive error. Characterizing these patterns could support the development of structured feedback and targeted educational interventions, particularly during the initial phases of program participation. Such an approach has the potential to reduce the likelihood of repeated errors and enhance overall reporting quality. Furthermore, these insights may serve as a valuable educational resource for institutions internationally that are planning to introduce or expand lung cancer screening initiatives.

## Conclusion

Robust multi-faceted quality assurance processes are required to maintain clinical quality as lung cancer screening initiatives evolve into population-level screening programs. For many radiologists, LCS reporting will be their first-hand independent exposure to screening which requires a refined and honed reporting approach compared to reporting for symptomatic patients. We have described a framework of how to track reporter performance in near real-time and then how to use those data metrics to iteratively improve reporter performance over time. Using empirical 2025-26 data we have also illustrated the potential for statistically-derived thresholds—both upper and lower—to complement the current NHSE fixed thresholds. We also provide a suggested standard operating policy to track performance, identify outliers, and help address any issues in reporting practice. We acknowledge that the near real-time framework described here has been developed and applied within a single national reporting network (the HLH), which reports a large but not exhaustive proportion of NHSE LCS examinations. It therefore complements, rather than replaces, the mandatory PERFECTS external quality assurance scheme, which remains the only program-wide QA covering every NHSE LCS reader. We propose that the elements described(quarterly referral-rate benchmarking with statistical thresholds, routine blinded double reading of a randomly sampled subset of green-RAG cases, structured outlier definition with rationale-based remediation, and granular individualized feedback) could form the basis of an NHSE/Department of Health and Social Care standardized near real-time QA framework that would be applicable across all LCS providers, alongside PERFECTS. We highlight areas of ongoing uncertainty and make suggestions for future work in the field.
